# Function and biotechnology of extremophilic enzymes in low water activity

**DOI:** 10.1186/2046-9063-8-4

**Published:** 2012-02-02

**Authors:** Ram Karan, Melinda D Capes, Shiladitya DasSarma

**Affiliations:** 1 Department of Microbiology and Immunology, University of Maryland School of Medicine, Baltimore, MD, USA; 2 Institute of Marine and Environmental Technology, University System of Maryland, Baltimore, MD, USA

**Keywords:** Extremophile, Extremozymes, Protein stability, Halophiles, Psychrophile, Cold activity, Organic solvent, Low temperature, High salinity, Biofuel, Bioenergy

## Abstract

Enzymes from extremophilic microorganisms usually catalyze chemical reactions in non-standard conditions. Such conditions promote aggregation, precipitation, and denaturation, reducing the activity of most non-extremophilic enzymes, frequently due to the absence of sufficient hydration. Some extremophilic enzymes maintain a tight hydration shell and remain active in solution even when liquid water is limiting, e.g. in the presence of high ionic concentrations, or at cold temperature when water is close to the freezing point. Extremophilic enzymes are able to compete for hydration via alterations especially to their surface through greater surface charges and increased molecular motion. These properties have enabled some extremophilic enzymes to function in the presence of non-aqueous organic solvents, with potential for design of useful catalysts. In this review, we summarize the current state of knowledge of extremophilic enzymes functioning in high salinity and cold temperatures, focusing on their strategy for function at low water activity. We discuss how the understanding of extremophilic enzyme function is leading to the design of a new generation of enzyme catalysts and their applications to biotechnology.

## Introduction

Enzymes are nature's biocatalysts endowed with high catalytic power, remarkable substrate specificity, and ability to work under mild reaction conditions. These unique features led to enzyme applications in competitive bioprocesses as one of the foremost areas of biotechnology research. Most enzymes are active within a defined set of standard conditions close to what is considered normal for mesophilic terrestrial organisms. However, much of the biosphere is extreme by comparison (e.g. cold oceans and dry, salty deserts). Not surprisingly, the biosphere contains a very large number of extremophilic microorganisms with enzymes capable of functioning in unusual conditions [1,2].

The discovery of thermostable DNA polymerases and their impact on research, medicine, and industry has underscored the potential benefits of enzymes from extreme environments [3]. Since that time, the biotechnological and industrial demand for stable enzymes functioning in harsh operational conditions has surged. A great deal of current effort is aimed at screening for new sources of novel enzymes capable of functioning in extreme conditions. The parallel development of sophisticated molecular biology tools has also enabled engineering of enzymes with novel properties using techniques such as site-directed mutagenesis, gene shuffling, directed evolution, chemical modifications and immobilization [4-6].

Microorganisms which grow in extreme conditions have been an important source of stable and valuable enzymes [1,7,8]. Their enzymes, sometimes called "extremozymes", perform the same enzymatic functions as their non-extreme counterparts, but they can catalyze such reactions in conditions which inhibit or denature the less extreme forms. Interestingly, some of the enzymes derived from extremophiles display polyextremophilicity, i.e. stability and activity in more than one extreme condition, including high salt, alkaline pH, low temperature, and non-aqueous medium [2,9-11]. A basic understanding of the stability and function of extremozymes under extreme conditions is important for innovations in biotechnology.

One of the underlying reasons for limited enzyme activity in extreme conditions is their effects on water structure and dynamics. When water activity is perturbed by extreme temperatures, high salinity, or other extreme conditions, normally structured liquid water may become limiting to enzymes, with deleterious consequences to enzyme structure and/or function. For example, at high salinity, water is sequestered in hydrated ionic structures, limiting the availability of free water molecules for protein hydration [12,13]. An analogous effect is felt by enzymes in cold temperatures due to the freezing of water molecules, forming structured ice-like lattices that are less available to interact with proteins [14]. Therefore, improved hydration characteristics in some extremozymes are critical for their function in their natural conditions. An interesting and potentially useful consequence of the hydration properties of such enzymes may be in extending their range of function to non-aqueous environments [5]. Enzymes capable of functioning in the presence of organic solvents may permit their use in some specialized applications, such as for catalysis of reactions using novel substrates. As a result, a better understanding of molecular mechanisms used by such extremozymes for improved solubility and hydration is of substantial biotechnological interest.

### Salt adapted enzymes

Water molecules are known to play a critical role in biological functions of proteins by binding to the surface and incorporating into the interior of protein molecules [15-18]. Water has a tendency to form ordered cages around hydrophobic groups on the protein surface [19]. Salt ions are known to disrupt the local water structure, diminishing the number of intermolecular hydrogen bonds [20-22]. High salt concentrations critically affect the solubility, binding, stability, and crystallization of proteins [23]. The interactions between proteins and protein subunits in solution are also altered by salts. The electrostatic interactions between charged amino acids are also perturbed with significant consequences for protein structure and function [24]. The effects depend on the chemical nature of the salts, generally following the position of ions in the Hofmeister series [25,26].

**Figure 1:**
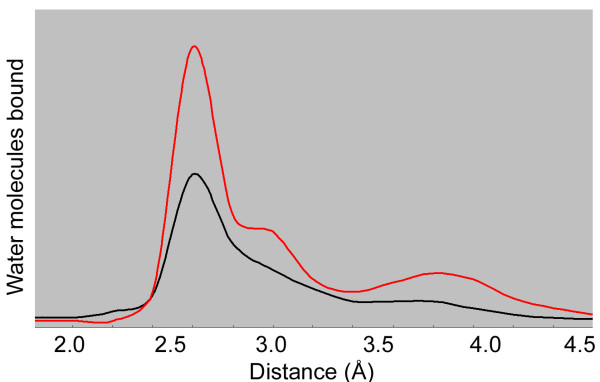
**Distribution of the water molecules near the protein surface predicted from high resolution structures (adapted from ref. 30)**. The relative number of water molecules versus distance is plotted for a halophilic glucose dehydrogenase enzyme active at low water activity (red) [30] and for non-halophilic enzymes (black). Multiple hydration layers may surround extremophilic proteins as a result of their ability to bind more tightly to water than non-extremophilic proteins.

Water is necessary for native structure, proper function, and to prevent aggregation of proteins. As salt ions inside the cell increases, water is removed from hydrophobic regions of protein surfaces, until proteins are no longer sufficiently hydrated [17,18,27] (Figure 1). Non-halophilic proteins are generally less able to compete with salts and lose their structure and activity at relatively lower ionic concentration. However, halophilic proteins are able to successfully compete with salt ions for hydration and maintain their functional conformation in the presence of high ionic concentration [28-30] (Table 1). This is especially true for proteins from halophilic microorganisms which use the salt-in mechanism for osmotic stabilization (e.g. halophilic archaea and some halophilic bacteria) [8]. Such halophilic proteins have evolved a specific set of molecular features that help them to compete with ions for water and maintain a stable hydration shell. In fact, in comparison to non-halophilic enzymes, halophilic enzymes are found to have multilayered hydration shells that are of considerably greater size and order (Figure 1).

**Table 1:** Extremophilic enzymes studied for function in low water activity

Name	Organism(s)	Method(s)	Reference(s)
**Salt adapted**			
α-amylase	*Halothermothrix *sp.	CD spectroscopy, sedimentation velocity, crystal structure	[59]
α-amylase	*Pseudoalteromonas *sp.	CD and fluorescence spectroscopy	[53]
Carbonic anhydrase	*Dunaliella *sp.	Crystal structure	[125]
Cysteinyl tRNA synthetase	*Halobacterium *sp.	Mutagenesis	[126]
Dihydrofolate reductase	*Halobacterium *sp.	Homology modeling	[127]
Dihydrofolate reductase	*Haloferax *sp.	Crystal structure	[41]
Dihydrofolate reductase	*Haloarcula *sp., *Halobacterium *sp., *Haloquadratum *sp., *Natrosomonas *sp.	Homology modeling	[38]
Dihydrolipoamide dehydrogenase	*Haloferax *sp.	Homology modeling, site-directed mutagenesis	[50]
DNA ligase	*Haloferax *sp.	Mutagenesis	[128]
DNA ligase	*Haloferax *sp.	Site-directed mutagenesis, CD, fluorescence and NMR spectroscopy	[35]
DNA ligase	*Haloferax *sp.	Homology modeling, CD spectroscopy	[26]
Esterase	*Haloarcula *sp.	Homology modeling, CD spectroscopy	[43]
Ferredoxin [2Fe-2S]	*Haloarcula *sp.	Crystal structure	[29]
Ferredoxin [2Fe-2S]	*Halobacterium *sp.	Fluorescence and CD spectroscopy	[48,129]
Glutamate dehydrogenase	*Halobacterium *sp.	Homology modeling	[130]
Glucose dehydrogenase	*Haloferax *sp.	Crystal structure	[30]
Glutaminase	*Micrococcus *sp.	Crystallization and X-ray crystallography	[131]
Malate dehydrogenase	*Halobacterium *sp.	Fluorescence spectroscopy	[132]
Malate dehydrogenase	*Halobacterium *sp.	Neutron scattering, ultracentrifugation and quasi-elastic light-scattering	[46]
Malate dehydrogenase	*Haloarcula *sp.	Densitometry and neutron scattering	[133]
Malate dehydrogenase	*Haloarcula *sp.	X-ray crystallography	[28]
Malate dehydrogenase	*Haloarcula *sp.	Site-directed mutagenesis	[134]
Malate dehydrogenase	*Haloarcula *sp.	Mutagenesis, crystal structure	[135]
Malate dehydrogenase	*Haloarcula *sp.	Crystal structure, neutron scattering	[23]
Malate dehydrogenase	*Salinibacter *sp.	Analytical centrifugation, CD spectroscopy	[136]
Malate dehydrogenase	*Haloarcula *sp.	Neutron diffraction, CD and neutron spectroscopy	[19]
Malate dehydrogenase	*Haloarcula *sp.	Neutron spectroscopy	[137]
Nucleoside diphosphate kinase	*Haloarcula *sp.	CD spectroscopy, crystal structure	[52]
Proliferating cell nuclear antigen	*Haloferax *sp.	Crystal structure	[42]
Protease	*Halobacillus sp*.	Fluorescence resonance energy transfer	[51]
TATA-box binding protein	*Pyrococcus *sp.	Analytical ultracentrifugation, isothermal titration calorimetry	[54]
TATA-box binding protein	*Pyrococcus *sp.	Site-directed mutagenesis, isothermal titration calorimetry	[55-57]
Xylanase	*Bacillus *sp.	Crystal structure	[138]

**Cold active**			
Adenylate kinase	*Bacillus *sp.	Crystal structure	[85]
Adenylate kinase	*Marinibacillus *sp.	Crystal structure, CD spectroscopy	[139]
Alkaline phosphatase	*Gadus *sp.	Fluorescence spectroscopy	[140]
Alkaline phosphatase	Antarctic strain TAB5	Site-directed mutagenesis	[84,141]
Alkaline phosphatase	*Vibrio *sp.	Mutagenesis, CD spectroscopy	[142]
Aminopeptidase	*Colwellia *sp.	Crystal structure	[143]
Aminopeptidase	*Colwellia *sp.	Differential scanning calorimetry, fluorescence spectroscopy	[144]
α-amylase	*Alteromonas *sp.	Crystal structure	[86]
α-amylase	*Pseudoalteromonas *sp.	Mutagenesis, differential scanning calorimetry, fluorescence spectroscopy	[145]
α-amylase	*Pseudoalteromonas *sp.	Differential scanning calorimetry, fluorescence spectroscopy	[79]
α-amylase	*Pseudoalteromonas *sp.	Matrix assisted laser desorption ionization time-of-flight mass spectrometry	[146]
α-amylase	*Alteromonas *sp.	Mutagenesis, crystal structure, molecular dynamics simulations	[147]
Aspartate aminotransferase	*Pseudoalteromonas *sp.	Homology modeling, CD and fluorescence spectroscopy	[148]
β-galactosidase	*Arthrobacter *sp.	Crystal structure	[149]
β-lactamase	*Pseudomonas *sp.	Crystal structure	[87]
Catalase	*Vibrio *sp.	Differential scanning calorimetry, fluorescence spectroscopy	[150]
Catalase	*Vibrio *sp.	Crystal structure	[151]
Chitinase	*Arthrobacter *sp.	Homology-modeling, mutagenesis, fluorescence spectroscopy	[77]
Chitobiase	*Arthrobacter *sp.	Differential scanning calorimetry	[152]
Citrate synthase	Antarctic bacterium DS2-3R	Crystal structure	[153]
Citrate synthase	*Arthrobacter *sp.	Site-directed mutagenesis	[154]
Citrate synthase	*Sulfolobus *sp.	Crystal structure	[155]
Citrate synthase	*Arthobacter *sp., *Pyrococcus *sp.	Homology modeling	[156]
Endonuclease I	*Vibrio *sp.	Crystal structure	[59]
Esterase	*Pseudoalteromonas *sp.	Fourier transform infrared spectroscopy, molecular dynamics simulation	[78]
Iron superoxide	*Pseudoalteromonas *sp.	Crystal structure, CD and fluorescence spectroscopy	[75]
Lipase	*Photobacterium *sp.	Crystal structure	[157]
Malate dehydrogenase	*Aquaspirillium *sp.	Crystal structure	[88]
Nitrate reductase	*Shewanella *sp.	Homology modeling	[158]
Pepsin	*Trematomus *sp.	Homology modeling	[159]
Protease	*Bacillus *sp.	Homology modeling, mutagenesis, CD spectroscopy	[160]
Protease	*Pseudomonas *sp.	Crystal structure	[161]
Protease	*Pseudoalteromonas *sp.	Homology modeling, CD, fluorescence spectroscopy	[162]
Protease	*Bacillus *sp.	Homology modeling, mutagenesis	[163]
Protease	*Vibrio *sp.	Site-directed mutagenesis	[164]
Protease	*Bacillus *sp.	Crystal structure	[165]
Protease	*Geomicrobium *sp.	Homology modeling, CD and fluorescence spectroscopy	[166,167]
Ribonuclease	*Shewanella *sp.	Site-directed mutagenesis, CD spectroscopy	[168]
Superoxide dismutase	*Aliivibrio *sp.	Crystal structure, differential scanning calorimetry	[93]
Subtilisin	*Bacillus *sp.	Site-directed mutagenesis	[169]
Triose phosphate isomerase	*Vibrio *sp.	Crystal structures, calorimetry	[98]

**Organic solvent active**			
Alcohol dehydrogenase	*Rhodococcus *sp.	Crystal structure	[119]
Protease	*Pseudomonas *sp.	Site-directed and random mutagenesis	[116,117]
Protease	*Pseudomonas *sp.	Homology modeling	[118]

**Figure 2:**
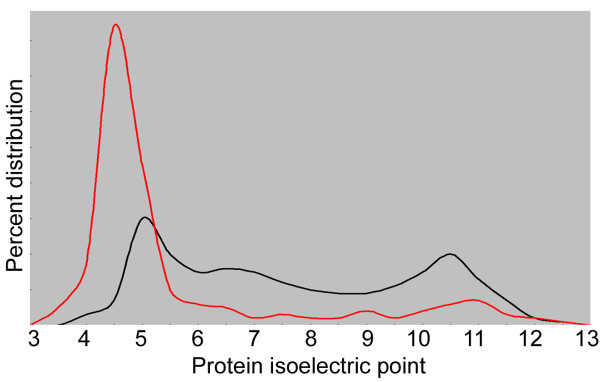
**Distribution of protein isoelectric point in halophilic and non-halophilic organisms predicted from genome sequences (adapted from ref. 13)**. The percent of all predicted proteins is plotted versus their calculated isoelectric points. The distribution of protein isoelectric points for the halophile *Halobacterium *sp. NRC-1 (red) is skewed towards acidic range while those of non-halophiles (black) have a broader distribution of isoelectric points with an average of neutrality in most cases.

In order to enhance activity in high salt concentrations, an increase in the number of charged amino acids, especially acidic residues at the protein surface, is observed in halophilic proteins [31-35] (Figure 2). Bioinformatic studies of the extreme halophile *Halobacterium *sp. NRC-1 and other species have shown that an increase in the number of acidic (glutamic acid, and to a lesser extent, aspartic acid) over basic residues is a general property of proteins predicted from the genomes of halophilic microorganisms [13,27] (Figure 2). Glutamate residues have superior water binding capacity over all other amino acids and are generally found in excess on the surface of halophilic proteins [15,16,28,30]. Acidic amino acids can constitute a high fraction of an individual protein, with up to 20-23% having been reported [36,37]. The negatively charged amino acids in halophilic proteins bind hydrated cations and help maintain a surface hydration layer, reducing their surface hydrophobicity, and contributing to mutual electrostatic repulsion [29,35,38]. These properties prevent aggregation at high salt concentrations [39].

**Figure 3:**
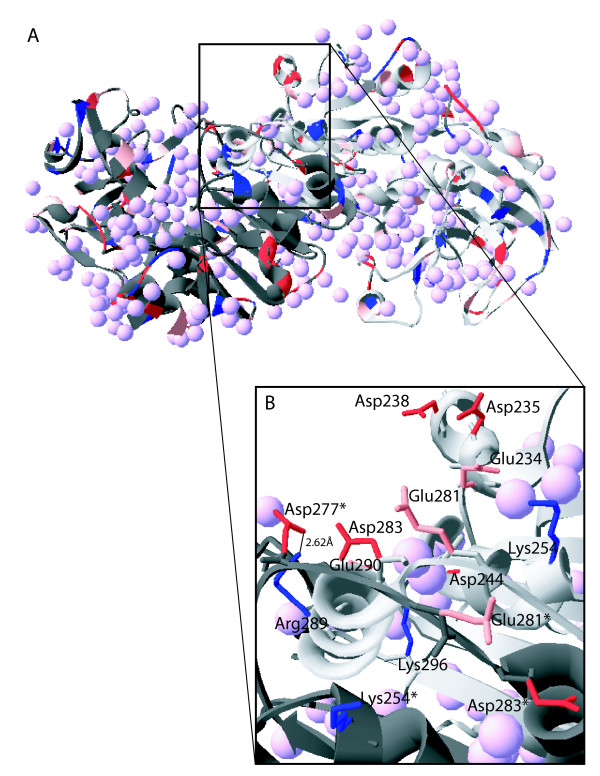
**Structural features of an extremophilic glucose dehydrogenase**. The protein structure (PDB ID:2B5V) [30] was downloaded from RCSB Protein Data Bank [123] and illustrated using DeepView Swiss-PdbViewer [124]. (A) Ribbon structure is shown with one subunit colored light gray and one subunit colored dark gray. Boxed region encompassing three α-helices of one subunit and two partial α-helices of the other subunit are shown in detail in part B. Acidic residues (aspartic acid and glutamic acid) are colored red and pink respectively, and basic residues (arginine and lysine) are colored dark blue and medium blue, respectively. Water molecules are colored light purple. (B) Expanded region showing a portion with side chains of exposed acidic residues and buried basic residues. Asterisk indicates residues of the dark gray subunit. An inter-subunit ion pair between Arg289 of one subunit and Asp277 of the other subunit is shown by a line labeled 2.62 Å, the distance between interacting atoms of the two residues.

X-ray and neutron diffraction structures have confirmed that the high content of acidic residues play significant roles in binding of essential water molecules and salt ions, preventing protein aggregation and providing flexibility to protein structure through electrostatic repulsion (Figure 3). For example, the structure of malate dehydrogenase from the extremely halophilic archaeaon *Haloarcula marismortui *received considerable attention from Mevarech and co-workers [40] and the group of Zaccai [19]. The presence of clusters of acidic residues has been observed in the crystal structure of dihydrofolate reductase (DHFR) and proliferating cell nuclear antigen (PCNA) from the extremely halophilic archaeaon *Haloferax volcanii *[41,42]. Crystal structure of the glucose dehydrogenase of the extremely halophilic archaeaon *H. mediterranei *has also contributed much information about halophilic adaptation and concluded that the surface of enzyme was predominantly acidic in nature and contributed to the halophilic characters of the enzyme [30]. In another study, Tadeo *et al*. [35] reported that by altering the amino acid composition at the protein surface, it is possible to modify the salt dependence of proteins and interconvert salt tolerant and non-tolerant proteins. Through the analysis of a large number of mutants, they concluded that the effect of salt on protein stability is largely independent of the total protein charge. In a recent study, a model of the recombinant esterase from *H. marismortui*, cloned and overexpressed in *Escherichia coli*, confirmed the enrichment of acidic residues and showed a high negative potential from clusters of aspartate and glutamate residues, with most acidic residues confined on the surface [43].

An interesting finding also apparent from the genome-wide bioinformatic analysis of predicted proteins was the deficit of protein surface lysine residues in halophilic proteins [13,27,33]. This observation is consistent with the reduction in hydrophobic surfaces in halophilic proteins, resulting in increased hydration at the protein surface. This prediction was confirmed directly via the structure of glucose dehydrogenase from the extreme halophile *H. mediterranei *solved by Britton *et al*. [30], where lysine residues on the enzyme surface have their side chains buried and better ordered than those from non-halophiles (Figure 3). In halophilic proteins, lysine residues are often replaced by arginines, likely due to the greater hydrophilicity of the guanidinyl side chain, with a substantial role in maintaining the active protein structure [11,30,35,38,44].

Halophilic proteins have also been found to contain a low content of bulky hydrophobic side chains on their surface, compared to non-halophilic proteins. The number of larger hydrophobic amino acid residues (phenylalanine, isoleucine, and leucine) is reduced compared to small (glycine and alanine) and borderline hydrophobic (serine and threonine) amino acid residues [27,31-34,38,45]. These findings are also consistent with increased flexibility, increased surface hydration, and reduced surface hydrophobicity of halophilic proteins.

In halophilic proteins, oppositely charged neighboring residues often interact to form salt bridges and these salt bridges play significant roles in protein folding, structure, and oligomerization (Figure 3). For example, the crystal structure of malate dehydrogenase from *H. marismortui *showed an increase in the number of salt bridges compared with the non-halophilic homologs, which enhanced enzyme stability at high salt concentrations [28]. This enzyme exists as a tetramer at high salt concentrations and dissociates into monomers as the salinity is reduced [46]. Similarly Madern *et al*. [47] have shown that isocitrate dehydrogenase from the halophilic archaeon *H. volcanii *exists as a dimer at high salt concentration but at low salt concentration it is irreversibly deactivated, due to dissociation of the dimer towards an inactive, partially folded monomeric species. *Halobacterium *sp. ferredoxin studied using CD and fluorescence techniques showed that the increase in salt concentration decreased electrostatic repulsion by ion binding, likely stabilizing oligomerization necessary for catalytic activity [48].

High salt concentrations generally enhance native conformation and functionality in halophilic proteins [31,49,50]. Salt concentrations may significantly affect the folding, conformation, subunit structure, and kinetics of halophilic proteins. Withdrawal of salt generally results in the gradual loss of protein structure and unfolding of halophilic proteins. Okamoto *et al*. [51] studied the role of salt on the kinetics of a high salt-active extracellular protease from the moderately halophilic bacterium *Halobacillus *sp. with fluorescence resonance energy transfer (FRET) and found that NaCl and other kosmotropic salts have a positive effect on catalytic activity of the enzyme. Their results suggested that these salts are excluded from the solvation shell of proteins because they have higher affinity for water than for the protein surface. Using far-ultraviolet circular dichroism (farUV-CD), Müller-Santos *et al*. [43] showed that in salt-free medium, an esterase from *H. marismortui *was completely unfolded and secondary structures appeared only in the presence of high concentrations of salt. The salt-dependent activity profiles of nucleoside diphosphate kinases from extremely halophilic archaea *Haloarcula quadrata *and *H. sinaiiensis*, have also been studied by farUV-CD spectroscopy, with salt-dependent oligomerization observed only for the latter [52]. Srimathi *et al*. [53] investigated a cold adapted amylase from the psychrophile *Pseudoalteromonas haloplanktis *by CD and fluorescence techniques. This cold-active amylase showed increased activity and improved folding at higher concentrations of salt similar to halophilic enzymes, indicating similar mechanisms of enhanced activity in both high salt and low temperature conditions.

Salt is also known to play a critical role in protein-DNA interactions. O'Brien *et al*. [54] studied the effect of salt on the thermodynamic-structural relationship of the binding of TATA box-binding protein (TBP) from *Pyrococcus woesei*, a moderately halophilic and hyperthermophilic organism, to its DNA binding site. This group hypothesized that uptake of cations and discharge of water accompanies protein-DNA complex formation. Subsequently Bergqvist *et al*. [55] used site-directed mutagenesis to change cation binding sites, i.e. negatively charged, acidic glutamate residues on the protein surface. Consistent with the hypothesis, they found that some of the mutants were able to convert the halophilic, relatively salt insensitive TBP into non-halophilic, salt sensitive variants [56,57].

While the underlying molecular mechanisms of halophilic protein function are still not fully understood, available studies have begun to shed considerable light on their strategies for adaptation to high salinity and relatively low water activity. Based on the many available studies, clustered surface negative charges, decreased hydrophobicity at the surface of the protein, and enrichment of salt bridges appear to be general strategies for improving the function of halophilic proteins in high salt, low water conditions. However, these mechanisms may not be universal [58,59], and additional research will continue to provide further insights into their function and activity. It is clear, however, that enzymes isolated from halophiles possess extraordinary structural and catalytic properties that allow function at low water activity. These exceptional biomolecules have great potential for applications to many biotechnological and industrial processes (Table 2).

**Table 2:** Extremophilic enzymes in biotechnology

Name	Organism	Activity			Application(s)	Reference(s)
				
		Salt	Cold	Organic solvent		
Amylase	*Pseudoalterimonas *sp.	+			*Saccharification of marine microalgae, saccharification of marine microalgae producing ethanol	[170]
Amylase	*Halococcus *sp.	+		+	Starch hydrolysis in industrial processes in saline and organic solvent medium	[171]
Amylase	*Streptomyces *sp.	+			Detergent formulations	[172]
Alcohol dehydrogenase	*Rhodococcus *sp.			+	*Enantioselective oxidation of sec-alcohol and the asymmetric reduction of ketones	[173]
Alkaline phosphatase	Antarctic bacteria strain HK47		+		*Radioactive end-labeling of nucleic acids	[174]
Alkaline phosphatase	Antarctic strain TAB5		+		*Dephosphorylation of DNA vectors	[84]
β-galactosidase	*Pseudoalteromonas *sp.		+		*Lactose hydrolysis	[175]
β-galactosidase	*Kluyveromyces *sp.			+	*Synthesis of galacto-oligosaccharides from lactose	[176]
β-galactosidase	*Arthrobacter *sp.		+		Lactose hydrolysis at low temperature, production of ethanol from lactose-based feedstock	[177]
β-galactosidase	*Bacillus *sp.			+	*Synthesis of N-acetyl-lactosamine	[178]
Chitinase	*Halobacterium *sp.	+		+	Oligosaccharide synthesis	[179]
Chitinase	*Virgibacillus *sp.	+			Bioconversion of chitin from fish, crab or shrimp; treatment of chitinous waste	[180]
Cholesterol oxidase	*Pseudomonas *sp.			+	Organic synthesis	[181]
Glutaminase	*Micrococcus *sp.	+			Flavor-enhancing in food industries, antileukaemic agent	[182]
Esterase	*Pyrobaculum *sp.			+	Organic synthesis	[183]
Esterase	*Pseudoalteromonas *sp.		+		Hydrolyzing esters of medical relevance	[184]
Lipase	*Candida *sp.		+		*Organic synthesis related to food/feed processing, pharmaceuticals or cosmetics	[185]
Lipase	*Rhizopus *sp., *Candida *sp.			+	Biodiesel production	[101-103]
Lipase	*Candida *sp.			+	Biodiesel production	[104,105]
Lipase	*Pseudoalteromonas *sp., *Psychrobacter *sp., *Vibrio *sp.		+	+	Detergent formulations and bioremediation of fat-contaminated aqueous systems	[186]
Lipase	*Staphylococcus *sp.		+		Detergent formulations	[187]
Lipase	*Psuedomonas *sp.			+	Biodiesel production	[106,107]
Lipase	*Salinivibrio *sp.	+			Detergent formulations and fatty acid degradations	[188]
Lipase	*Psuedomonas *sp.			+	Solvent bioremediation, biotransformations and detergent formulations	[189]
Lipase	*Marinobacter *sp.	+		+	*Hydrolysis of fish oil into free eicosapentaenoic acid	[190]
Nuclease	*Micrococcus *sp.	+			*Production of the flavoring agent 5'-guanylic acid	[191]
Pectinase	*Pseudoalteromonas *sp.		+	+	Enhancing extraction yield, clarification, and taste of fruit juices	[192]
Protease	*Halobacterium *sp.	+		+	*Peptide synthesis	[193,194]
Protease	*Pseudomonas *sp.			+	*Synthesis of N-carbobenzoxy-L-arginine-L-leucine amide, N-carbobenzoxy-L-alanine-L-leucine amide and aspartame precursor	[195,196]
Protease	*Pseudomonas *sp.			+	Peptide synthesis	[197]
Protease	*Bacillus *sp.		+		*Cleansing of contact lenses	[198]
Protease	*Natrialba *sp.	+		+	*Synthesis of tripeptide Ac-Phe-Gly-Phe-NH_2_	[199]
Protease	*Halobacterium *sp.	+			*Fish sauce preparation	[200]
Protease	*Geomicrobium *sp.	+		+	Peptide synthesis, detergent formulations	[201,202]
Xylanase	*Pseudoalteromonas *sp.		+		*Baking industry for increasing loaf volume	[203,204]
Xylanase	*Bacillus *sp.	+			Xylan biodegradation in pulp and paper industry	[205,206]

* applications established in laboratory and/or industry

### Cold active enzymes

Like high salinity, cold temperatures also critically affect the properties and structures of enzymes as well as the surrounding water. Cold temperatures affect the dynamic activity of bulk water as well as the spheres of hydration surrounding the protein surface [60]. Since water acts as a lubricant, easing the essential peptide amide-carbonyl hydrogen bonding dynamic, the effects of water are highly dependent on the temperature [61-63]. Dependence of the strength of aqueous hydrogen bonds on temperature is neither linear nor monotonic but unimodal, with the maximum density for pure water at approximately 4°C. Broken hydrogen bonds are found in high-density water while strong networks of hydrogen bonds are found in low-density water [63]. Deficiency or disturbance of hydrogen bonds and water networks around the protein may be linked to the loss of biological activity and protein denaturation at low temperatures [64].

As temperature decreases, the water molecules surrounding the protein surface become more ordered and thereby less associated with the protein, eventually pushing the system equilibrium toward the unfolded or denatured state. This change in protein structure is driven by an increase in hydration energies of non-polar groups at lower temperature [65,66]. The hydration energies of cold-active enzymes are generally less affected by lower temperature, and their lower inherent surface hydrophobicity is less sensitive than mesophilic proteins, keeping their structures more intact [67]. In effect, cold active proteins are able to hold on more tightly to the available water, similar to salt adapted proteins.

Temperature and viscosity of the medium are inversely related, with viscosity halved when the temperature is reduced from 37°C to 0°C [68]. Low temperatures therefore reduce the speed of reactions at least in part due to the strong effect of temperature on viscosity of the medium [68-71]. Based on biophysical considerations, reaction rates are predicted to decrease 2-3 fold for every 10°C decrease in temperature, according to the Arrhenius equation [72]. As a result, the effect of reduced temperature on enzyme activity is very significant, and the design of cold-active enzymes must have some structural adaptations to maintain the level of 'breathing' necessary for catalysis [73,74].

Studies of cold active enzymes have suggested that both an increase in interactions with the solvent and an increase in structural flexibility contribute to maintaining catalytic activity at lower temperatures [14,73,75-83] (Table 1). Aurilia *et al*. [78] used Fourier transform infrared spectroscopy (FTIR) and molecular dynamics simulations to investigate a cold active esterase from *P. haloplanktis *and found that the flexibility of the protein loop near the active site plays a crucial role in its function. An α-amylase from the same organism was investigated by D'Amico *et al*. [79] and shown using differential scanning calorimetry and fluorescence spectroscopy to have high conformational flexibility and electrostatic potential at the protein surface, which lowers activation energy for hydrolysis. The temperature dependence of alkaline phosphatase from the Antarctic strain TAB5 was studied by selection of thermostable and thermolabile variants. The study showed that cold activity is sensitive to slight changes in the protein sequence, particularly in residues located within or close to the active site of the enzyme [84].

There are relatively few solved three-dimensional structures of cold-active proteins compared to mesophilic or thermophilic proteins. While overall structures of cold adapted proteins are generally almost identical to mesophilic and thermophilic homologs [76], variations that are observed confirm an overall increase in protein flexibility and solvent interactions. For example, the crystal structures of cold active superoxide dismutase from *P. haloplanktis *and *Aliivibrio salmonicida *were compared with the mesophilic homolog from *E. coli*. Both cold-active superoxide dismutases showed an increased flexibility of the active site residues with respect to their mesophilic homologue [75]. Bae and Phillips [85] compared the crystal structures of adenylate kinases from the psychrophile *B. globisporus *and the mesophile *B. subtilis *with the thermophilic *B. stearothermophilus *enzyme. They concluded that the maintenance of proper flexibility is critical for the cold active proteins to function at their environmental temperatures. Similarly the crystal structure of *Alteromonas haloplanctis *α-amylase and *Pseudomonas fluorescens *β-lactamase showed a decrease in the number of hydrogen-bonds favoring more flexibility in these cold-active enzymes [86,87]. The crystal structure of a cold active malate dehydrogenase from *Aquaspirillium arcticum *showed similar features to be responsible for cold activity, including an increased flexibility around the active site region, more favorable surface charge distribution for substrate and cofactor interactions, and reduced intersubunit ion pair interactions [88].

A bioinformatic study of amino acid contacts that differ between proteins adapted to different temperatures, which included nearly 400 psychrophilic proteins, found that interactions with the solvent at the protein surface play an important role in temperature adaptation [89]. Additional bioinformatic and experimental studies have also suggested that the temperature-dependent activity of cold active enzymes may be altered by changing the amino acid composition, especially the overall protein charge, decreasing the hydrophobicity in the core of the enzyme, or decreasing the number of hydrogen bonds, salt bridges, or bound ions at the surface [72,90-92].

Amino acids present on the protein surface of cold active enzymes have been shown to play critical roles in both activity in cold and in high salinity, with increased activity and improved folding at higher concentrations of salt [53,59]. Moreover, the crystal structure of a cold-active iron superoxide dismutase from the *A. salmonicida *also showed an increase in the net negative charge on the surface of cold-active iron superoxide dismutase [93]. These findings and others [94-97] suggest that solvent interactions of cold active enzymes display remarkable similarity to salt adapted enzymes.

While the adaptive mechanisms of cold active proteins are still under investigation, the best understood mechanisms include increased conformational flexibility at the expense of stability and enhanced interactions with the solvent. To increase flexibility, many structural modifications and the disappearance of discrete stabilizing interactions (electrostatic interactions and an improved interaction of surface side chains with the solvent) have been observed. While nearly all cold active enzymes studied to date have shown these tendencies, there are examples of unusual enzymes, *e.g*. enzymes displaying rapid kinetics, which display temperature independent characteristics. Triosephosphate isomerase from *Vibrio marinus *is an example of a temperature independent enzyme [98]. The number of cold active proteins studied is expanding and additional future research including comparisons with closely related mesophilic homologs will provide further insights into enzymology at low temperature. The novel properties of cold active enzymes are likely to be valuable for a variety of applications in biotechnology and industry [99].

### Enzyme function in organic solvents

One of the most useful outcomes of a better understanding of enzyme-solvent interactions is the potential engineering of new and more effective catalysts functioning in non-aqueous environments. Such enzymes may be useful for both biofuel and bioenergy applications, where large quantity of ethanol or other organic solvents are produced [100-107], and for synthetic chemistry, especially when catalysis of desired chemical reactions requires the presence of organic solvents [108,109]. Organic solvents in the reaction mixture increase the solubility of hydrophobic substrates, and have the potential to improve the kinetic equilibrium and increase the yield and specificity of the product. However, limiting the disruption of molecular interactions in enzymes by organic solvents and the concomitant loss of activity remains a significant challenge.

A main factor responsible for loss of enzyme activity in organic solvents is the loss of crucial water molecules [109,110]. The low water content restrains protein conformation mobility and affects K_m _and V_max _values [111]. This rigidity increases resistance to thermal vibrations and reduces the enzyme-substrate interactions, leading to a reduced catalytic rate [112,113]. Loss of water can also disrupt hydrogen bond formation between protein subunits on the exterior surface and active site interactions in the interior of proteins may be weakened. The presence of organic solvents can cause disruption of the forces important to the hydrophobic core due to the increased hydrophobicity of the medium. Low water activity may limit diffusion of substrates and stabilize the ground state of the enzyme or change the enzyme conformation altogether. Enzymes in non-aqueous systems can be active provided that the enzyme surface and the active site region are well hydrated [114].

The polarity of organic solvents is the most important factor in the balance between stabilization and inactivation of enzymes. Co-solvents systems (water plus water-miscible organic solvents), organic aqueous biphasic systems (water plus water-immiscible organic solvents), nearly anhydrous systems, and reverse micelles may be the result of addition of organic solvents with water. The relative proportion of organic solvent and water depends on the miscibility of the components [109-115]. Highly polar, miscible organic solvents may strip the hydration layer from the enzyme surface, affecting enzyme flexibility and catalytic activity. Hydrophobic solvents, in contrast, may form a two-phase non-homogeneous system, leaving the hydration shell of the protein intact. However, they may sequester substrate away from the enzyme, depending on solubility and partitioning between phases. For improved activity in two-phase systems, a microenvironment or surface where favorable conditions (high enzyme activity, high substrate concentration, and low product solubility) driving high reaction rates may be desirable.

Published studies of the mechanism of adaptation of enzymes to function in organic solvent are relatively few. Ogino *et al*. [116,117] investigated the mechanism of organic solvent tolerance in a *Pseudomonas aeruginosa *PST-01 protease by site-directed and random mutagenesis. They reported that the disulfide bonds and amino acid residues located on the surface of the molecule play important roles in organic solvent stability of the enzymes. The structural analysis of organic solvent effects on a protease from a similar *P. aeruginosa *strain also identified two disulfide bonds as well as a number of hydrophobic clusters at the protein surface. These hydrophobic patches and disulfide bonds were proposed to be responsible for the solvent-stable nature of the enzyme [118]. Recently, Karabec *et al*. [119] solved the crystal structure of alcohol dehydrogenase from *Rhodococcus ruber *DSM 44541 and suggested that salt-bridges play a significance role in the stability of this enzyme in non-aqueous media.

Organic solvent mediated enzymatic reactions have many advantages over aqueous enzymatic reactions: (i) increased solubility of apolar substrate and alteration in substrate specificity, (ii) enhanced regio-and stereo-selectivity, (iii) absence of racemization, (iv) lack of requirement of side chain protection, (v) reduced water activity altering the hydrolytic equilibrium, (vi) elimination of microbial contamination, and (vii) suppression of unwanted water-dependent side reactions [108,109,114,115]. Additionally, enzymes in organic solvents tend to be more rigid than in water (due to increased electrostatic and hydrogen bonding interactions among the surface residues of enzyme) and provide the possibility of techniques such as molecular imprinting [109]. In molecular imprinting, the enzyme solution is freeze-dried with a ligand ("imprinter") that locks the enzymes into a catalysis favorable condition during lyophilization and enhances the enzyme activity in organic solvents [120]. In some cases, it has been found that molecular imprinting increases enzyme activity in organic solvents when co-lyophilized with an inorganic salt such as KCl. KCl prevents the reversible denaturation of proteins and produces a strong additive activation effect during the drying process [121]. Among the disadvantages of non-aqueous organic enzyme catalysis, the loss of catalytic activity is the most common. Many enzymatic reactions are also susceptible to substrate and/or product inhibition, and expensive natural cofactors for enzyme activity may be required for full activity. Finally, labor and cost-intensive preparation of biocatalysts and enzymes may require narrow operation parameters, limiting the value of some enzymes that may be active in the presence of organic solvents [109,122]. However, the potential for organic solvent mediated enzyme catalysis to enable desirable biotechnological aims remains a major motivating factor for additional research.

## Conclusion

Extremophilic salt adapted and cold active enzymes have expanded our understanding of enzyme stability and activity mechanisms, protein structure-function relationships, and enzyme engineering and evolution. The still emerging understanding of protein-solvent interactions are likely to aid in development of new catalysts for use in novel synthetic applications, including enzymes operating in low water activity and organic solvents, and in the development of efficient catalytic systems active in organic solvents for applications in bioenergy and biotechnology.

## Competing interests

The authors declare that they have no competing interests.

## Authors' contributions

The manuscript was drafted by RK and revised and finalized together with MDC and SD. All authors have read and approved the final manuscript.
